# Nutritionally-Induced Catch-Up Growth

**DOI:** 10.3390/nu7010517

**Published:** 2015-01-14

**Authors:** Galia Gat-Yablonski, Moshe Phillip

**Affiliations:** The Jesse Z and Sara Lea Shafer Institute for Endocrinology and Diabetes, National Center for Children’s Diabetes, Schneider Children’s Medical Center of Israel, and Felsenstein Medical Research Center, Petach Tikva 49100, and Sackler School of Medicine, Tel Aviv University, Tel Aviv, 69978, Israel; E-Mail: mosheph@post.tau.ac.il

**Keywords:** growth, growth plate, nutrition, catch-up growth

## Abstract

Malnutrition is considered a leading cause of growth attenuation in children. When food is replenished, spontaneous catch-up (CU) growth usually occurs, bringing the child back to its original growth trajectory. However, in some cases, the CU growth is not complete, leading to a permanent growth deficit. This review summarizes our current knowledge regarding the mechanism regulating nutrition and growth, including systemic factors, such as insulin, growth hormone, insulin- like growth factor-1, vitamin D, fibroblast growth factor-21, *etc.*, and local mechanisms, including autophagy, as well as regulators of transcription, protein synthesis, miRNAs and epigenetics. Studying the molecular mechanisms regulating CU growth may lead to the establishment of better nutritional and therapeutic regimens for more effective CU growth in children with malnutrition and growth abnormalities. It will be fascinating to follow this research in the coming years and to translate the knowledge gained to clinical benefit.

## 1. Introduction

Numerous genetic and environmental factors may affect growth, but malnutrition, marked by various nutrient deficiencies, is considered a leading cause of failure to thrive and growth attenuation in children. According to the UNICEF-WHO-The World Bank: 2012 joint report, linear growth restriction or stunting (height below minus two standard deviations from the median height for age of the reference population) due to chronic malnutrition affects an average of 25% of all children younger than five years worldwide [1]. Although these numbers reflect a decrease in the prevalence of stunting, the actual number of affected children, most of them in Asia (56%) and Africa (36%), still amounts to a vast number of 162 million children.

**Figure 1:**
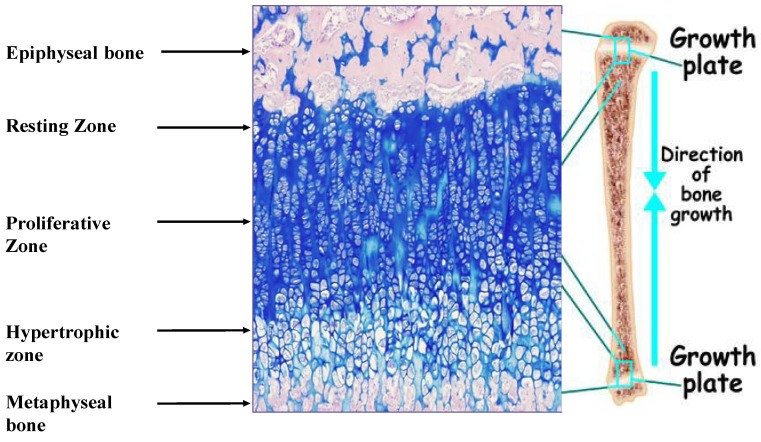
Epiphyseal growth plate of male Sprague Dawley rat (34 days old) stained with hematoxylin/eosine/Alcian Blue. Magnification, 100×. The different zones of the growth plate are marked.

Linear growth of the appendicular skeleton is the product of a cascade of events that take place in the cartilaginous growth center of the long bones, termed the epiphyseal growth plate (EGP) (Figure 1). It is controlled by complex interactions among hormones, local growth factors and components of the extracellular matrix (ECM). The process is driven by the chondrocytes. It begins with the proliferation of resting early chondrocytes located at the most epiphyseal end of the EGP, followed by their alignment in columns parallel to the long axis of the bone. The cells then undergo a period of high secretory activity, depositing the cartilage ECM components, including collagens, proteoglycans and other materials, until their maturation into hypertrophic chondrocytes. The transition from cell proliferation to hypertrophy takes place in the pre-hypertrophic zone located in the middle of the EGP. Several important regulatory molecules, such as Indian hedgehog (Ihh) and the parathyroid hormone-related protein (PTHrP) receptor, are expressed specifically in this region [2]. Once the hypertrophic cells cease dividing, they increase in volume by 5–10-fold owing to the ingress of water and begin to secrete ECM, consisting of collagen type X, as well as small matrix vesicles that serve as centers of mineralization. Thereafter, the chondrocytes undergo programmed physiological cell death [3], with calcification of the ECM. Blood vessels, osteoclasts and osteoblasts are now able to enter the ECM, and the cartilage scaffold is replaced with bone tissue. The reorganization of the ECM is crucial to the proper development of the EGP [4].

Good nutrition ensures the availability of the proper “building blocks” for growth, including proteins, lipids and carbohydrates. Accordingly, fasting impairs the rate of longitudinal bone growth and reduces the length of the EGP [5,6,7]. Animal studies clearly demonstrated the deleterious effects of protein energy malnutrition on linear growth, but in humans, it is somewhat difficult to dissociate the effect of the nutritional and other environmental factors or to ascertain the irreversibility of the nutritional damage. In the presence of infection, protein energy malnutrition induced greater loss of nutrients or led to metabolic alterations [8]. Children with marasmus (a form of malnutrition caused by long-lasting insufficient caloric intake) and kwashiorkor (a form of malnutrition caused mainly by insufficient protein consumption, due to either low quality or low quantity protein consumption) have been found to have significantly lower body weight and height than healthy subjects [9,10]. In children with idiopathic short stature from developed countries, where plenty of variable food is available, calorie intake was positively correlated with growth velocity, both before growth hormone (GH) treatment and during the first year of GH treatment [11]. Children with eating disorders from developed countries were on average shorter than controls; this effect was independent of age of onset [12].

In most cases, when the malnutrition is resolved, spontaneous catch-up (CU) growth occurs. CU growth is defined as “height velocity above the normal statistical limits for age and/or maturity during a defined period of time following a transient period of growth inhibition” [13]. It culminates with the individual reaching his/her original, pre-growth-restriction growth curve. Excellent examples of nutrition-induced CU growth are found in children with celiac disease who exhibit a remarkable growth spurt shortly after the onset of a gluten-free diet [13]. Apparently, in states of growth restriction, the EGP is able to conserve much of its growth capacity until conditions improve, enabling CU growth [14]. Two hypotheses have been suggested to explain this phenomenon. According to the first, a still unidentified neuroendocrine factor “compares” the individual’s actual size with his/her chronologically-expected size and adjusts the growth rate accordingly [15]. The second, now commonly accepted, hypothesis is based on the normal process of senescence, whereby growth ceases with increasing age due to a decrease in the overall height of the EGP, concomitant with a reduction in the number of resting, proliferating and hypertrophic chondrocytes, as well as the size of the hypertrophic chondrocytes [16]. The hypothesis suggests that malnutrition, elevated glucocorticoid levels [17], hypothyroidism [18] and tryptophan deficiency [17] can all slow senescence in the growth-arrested EGP [19], keeping the EGP in a “younger phase” until conditions for growth are regained. The senescence hypothesis is supported by the above-mentioned elegant studies conducted by Baron and colleagues [17,18,19].

**Figure 2:**
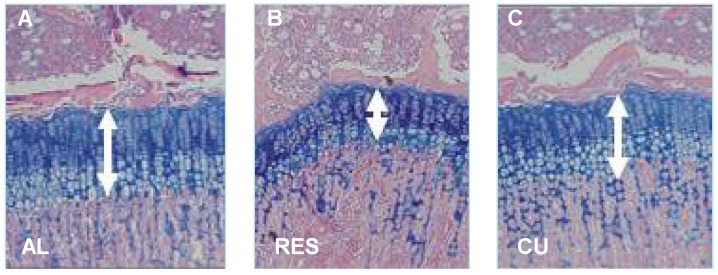
Effect of food restriction and re-feeding on the height of the EGP. Twenty-four-day-old male SD rats were allowed to eat *ad libitum* (AL), subjected to 40% food restriction for 11 days (RES) or subjected to 10 days of food restriction followed by one day of re-feeding *ad libitum* (CU). The arrows indicate the height of the EGP. Magnification, 40×.

To further investigate the mechanism controlling nutritionally-induced CU growth postnatally, we established a model wherein young rodents were subjected to 40% food restriction for 10 days, followed by unrestricted re-feeding [7,20,21]. The results showed that, on average, the food-restricted rats gained only 1.2 g/day as opposed to 6.5 g/day in rats fed *ad libitum.* When the food restriction was removed, the rats rapidly gained weight; with the largest increase (15.1 g) occurring on the first day. The height of the EGP, measured from the reserve zone to the ossification front of the metaphyseal bone, was significantly reduced in the food-restricted rats and increased significantly already after one day of food restoration. Bone length increased significantly seven days later (Figure 2) [7].

Despite enormous efforts by pediatric endocrinologists, dieticians and research scientists to expand our understanding of the interaction of nutrition and linear growth in children, the exact mechanism whereby the body signals the EGP to grow or attenuate growth is still unclear. The aim of the present work was to review the systemic and local mechanisms involved with linear growth during malnutrition and CU growth at the level of the EGP in the postnatal growth period.

## 2. Systemic Factors in Malnutrition and CU Growth

Negative energy balance leads to reduced plasma levels of insulin, insulin-like growth factor-1 (IGF-1), thyroid hormone, leptin and sex hormones (not discussed in this review) and increases the levels of glucocorticoids (GC)s and IGF binding protein (IGFBP)-1 and -7. Other systemic factor that may be involved are fibroblast growth factor 21 (FGF-21) and vitamin D, which may also be affected by nutrition. All of these factors have a direct effect on linear growth, and thus, the consequence of nutritional restriction is a reduction in growth and body size (Table 1). However, the EGP is able to conserve much of its growth capacity until conditions improve, enabling CU growth [14].

### 2.1. Insulin

Insulin, a 51-amino acid β-cell-specific hormone, is secreted from the pancreas in response to increased glucose levels and binds to its receptors on peripheral cells and tissues, making it possible for glucose to assimilate into the cells. Insulin was the first hormone to be identified in the control of body weight [22]. Its essential role in normal intrauterine growth is suggested by findings of severe intrauterine growth retardation (IUGR) in babies with pancreatic agenesis [23] or mutations in the insulin receptor gene [24,25] and in studies of leprechaunism, a congenital disorder caused by a mutation in the insulin receptor gene, which is characterized by insulin resistance, fasting hypoglycemia and severe pre- and post-natal growth restriction [26]. The outcome may be attributed to an insufficient energy supply to the cells or a lack of insulin activity on chondrocytes of the EGP. Other evidence is derived from studies of mutations in the gene controlling the production of the enzyme, glucokinase, which catalyzes the rate-limiting step in glycolysis and serves as a pancreatic β-cell glucose sensor. Affected children have altered glucose sensing and decreased insulin secretion and are approximately 500 g smaller than their unaffected siblings [27]. Accordingly, in children with chronic, poorly-controlled type 1 diabetes, the lack of adequate insulin levels may lead to growth failure. However, in these cases, the growth failure is usually modest and is probably due to a combination of calorie wasting, chronic acidosis and increased glucocorticoid production, also characteristic of other chronic diseases [28]. In most instances, there is no correlation between glycemic control and skeletal growth, and many children with apparently marginal control appear to grow well.

**Table 1:** Hormones and growth factors that are specifically affected by the nutritional status.

Hormone	Affected by food restriction	Effect on growth
Insulin	Reduced	Stimulates growth
Growth Hormone	Reduced (rats and mice)/increased (humans, rabbits, sheep, cows and pigs)	Stimulates growth
Insulin like growth factor 1	Reduced	Stimulates growth
IGFBP-1	Increased	Inhibits growth
Leptin	Reduced	Stimulates growth
Glucocorticoids	Increased	Inhibits growth
Thyroid hormones	Reduced	Stimulates growth
FGF21	Reduced/increased	Inhibits growth
Vitamin D	Reduced	Required for proper growth, inhibits chondrocyte proliferation at high concentrations
Sex hormones *	Reduced	Stimulates growth (testosterone), hastens EGP closure (estrogen) *

* sex hormones are not discussed in this review.

Much attention has been directed to insulin in investigations of CU growth, mostly because the accelerated postnatal CU growth has been associated with an increased risk of obesity, type 2 diabetes and other features of metabolic syndrome in adulthood. Barker and his colleagues suggested “the thrifty phenotype hypothesis”, whereby deficient food supply to the fetus may cause an aberrant programming, which includes circulatory adjustment and insulin resistance in liver and muscular tissue in order to spare the brain. Additional studies also suggested an increased risk of late-onset metabolic diseases in adults who underwent a period of rapid CU growth as children (reviewed by [29]). Indeed, there is evidence suggesting that malnutrition in early life impairs the expression of several insulin-signaling proteins [30] and that the development of insulin resistance is closely correlated with a higher body mass index and postnatal CU height in small for gestational age (SGA) children [31]. However, other studies yielded no difference in insulin sensitivity between food-restricted and re-fed animals [32]. Thus, the insulin sensitivity and metabolic outcome may depend on the modes of restriction and re-feeding.

### 2.2. Growth Hormone and Insulin-Like Growth Factor-1

During childhood, linear growth is predominantly regulated by growth hormone (GH), a 191-amino acid polypeptide synthesized and secreted by somatotrophs of the anterior pituitary gland. GH also exerts metabolic effects on numerous tissues and organs. Its release from the pituitary is regulated by a complex interplay of hypothalamic, pituitary and circulating factors. GH affects growth directly, by binding to its receptors in the EGP, and also indirectly, via insulin-like growth factor 1 (IGF-1), a 70-amino acid peptide produced mainly in the liver. IGF-1 is both the main mediator of GH action and a GH-independent growth factor, especially *in utero*, stimulating cell proliferation and differentiation and protecting cells from apoptosis. Fetuses with deficient IGF-1 production or a defect in the IGF-1 receptor show significant growth delay, whereas fetuses with GH deficiency do not [33]. IGF-1 acts through its receptor, IGF-1R, which is expressed on all tissues, including cells of the EGP, where IGF-1 acts in a spatial-dependent manner [34,35].

Studies of mice with targeted mutagenesis of the genes encoding IGF-1 and IGF-1R found that IGF-1^−/−^ mice had a birth weight of 60% of their wild-type littermates, and IGF-1R^−/−^ mice had a birth weight of 45% of normal. The IGF-1R^−/−^ mice all died immediately postpartum from respiratory failure [36]. In humans, inactivation mutations in the IGF-1 gene are associated with growth restriction [25,37], and all mutations in the IGF-1R gene identified so far are associated with severe IUGR (length deficit of −0.3 to −5.8 standard deviation score (SDS) depending on the specific mutation) and short stature [25,33,38,39,40,41,42,43,44].

GH and IGF-1 concentrations are known to be responsive to changing nutritional status [45,46]. Fasting induces a GH-resistant state: it increases serum GH levels in humans, rabbits, sheep, cows and pigs, but reduces serum GH levels in mice and rats. Nevertheless, in all animals examined, IGF-1 levels were reduced. On immunohistochemistry study of food-restricted mice, a reduced positive staining with anti-GH receptor (GHR) and anti-IGF-1R antibodies in the EGP sections was noted [47]. Upon western immunoblot, there was a marked reduction of IGF-1R in the EGP [48], indicating that the EGP is rendered GH- and IGF-1-resistant at the receptor level during food restriction [7]. It is possible that the decrease in plasma IGF-1 and local IGF-1R and GHR is part of the body’s effort to adapt by shunting calories away from nonessential processes, including growth, during periods of malnutrition [49]. Interestingly, levels of IGF-1 and IGF-1R increased after only one day of re-feeding [21,48], enabling CU growth. These results are in line with the clinical observation that unlike well-fed children, malnourished children do not respond well to GH treatment [50], and re-feeding is required to achieve an optimal response.

IGFs circulate in the plasma tightly bound to the specific IGF binding protein (IGFBP) family, which extend the serum half-life of IGF peptides, transport the IGFs to target cells and modulate the interaction of the IGFs with their surface membrane receptors. There are six proteins with high affinity to IGFs (IGFBP1-6) and others with lower affinity. IGFBP-1 binds IGF-1 with high affinity, inhibiting its binding to the receptor, while IGF-1 suppresses IGFBP-1 protein levels, probably at the transcription level [51]. In humans, IUGR is correlated with high levels of serum IGFBP-1, and in rats, maternal overexpression of IGFBP-1 during pregnancy was clearly associated with neonate growth retardation [52]. In transgenic mice that overexpressed human IGFBP-1, growth retardation was observed together with pleiotropic defects of several skeletal units with reduced mineralization and dysmorphic bones [51].

IGFBP-1 levels are related to changes in glucose and insulin [53]; IGFBP-1 production is suppressed by insulin, while it is significantly increased by food restriction [54], energy restriction [55] and dietary restriction of proteins [55] or specific amino acids in rats [56], further reducing the bio-availability of IGF-1. Interestingly, this was not observed when the diets differed in carbohydrate and fat content [57]. It was shown that leucine deprivation causes phosphorylation of IGFBP-1 at discrete sites that markedly enhance IGF-1 affinity, stabilizing the IGF-1-IGFBP-1 complex, leading to further inhibition of IGF-1-stimulated cell growth [58].

IGFBP-7 (mac 25/angiomodulin/IGFBP-related protein 1), is a secreted, 27-kDa protein that binds IGF-1 with relatively weak affinity [59]. It plays a multifunctional role in regulating cellular proliferation, adhesion and angiogenesis and was shown to be involved in senescence and apoptosis. Findings of IGFBP-7 downregulation in several tumor cell lines and its high expression in senescent cells suggest that it may also exert tumor-suppressive or anti-proliferative activity in normal tissue [59]. IGFBP-7 apparently has a negative effect on growth, as indicated by findings that it increased in the EGP with aging [60] and in association with growth attenuation [7]. Interestingly, in our food restriction CU model, we showed that already after one day of re-feeding, the level of IGFBP-7 was reduced back to normal, further supporting the notion that the EGP returns to an active growth phase.

### 2.3. Leptin

Leptin, a hormone predominantly produced by adipocytes [61], was originally described as a circulating hormone involved in feeding behavior and energy homeostasis [62,63]. Only later was it found to have numerous peripheral effects, including bone growth and remodeling [20,47,64,65,66,67,68,69]. In children, linear growth apparently starts only after they attain at least 85% weight for length, and periods of rapid growth, such as fetal life [70] and adolescence [71,72], require a certain minimal level of leptin. These findings are compatible with a role of leptin as a metabolic signaling agent connecting adipocyte tissues with the GH axis. A direct link between leptin and linear growth was suggested by findings that leptin administration to the leptin-deficient *ob*/*ob* mice corrected their metabolic abnormalities and also led a significant increase in femoral length [73,74]. Leptin was shown to directly stimulate GH secretion [75,76,77,78] and increase the level of IGF-1 receptor [66,79]. Data on the effect of leptin on bone mass and length are conflicting. Ducy *et al.* [80] showed that hypothalamic administration of leptin decreased bone mass in *ob*/*ob* mice by inhibiting bone formation through a pathway involving increased sympathetic signaling. By contrast, Hamrick *et al.* [81] showed that in growing *ob*/*ob* mice, hypothalamic leptin gene therapy increased bone length and total bone mass. In a recent paper, Turner and colleagues concluded that leptin signaling affects bone formation and resorption, primarily via a peripheral route [69]. Although leptin deficiency in mice is associated with impaired linear growth, Montague *et al.* [82] described a family with a mutation in leptin in which the index patient and her affected cousin were both tall, and Farooqi [83] showed that children with a congenital mutation in the leptin receptor had normal linear growth. Thus, the effect of leptin may differ between rodents and humans.

We found that normal mice treated with repeated subcutaneous leptin injections have longer tibia than pair-fed controls. Leptin stimulation of the EGP was balanced, positively affecting both proliferation and differentiation, so that the ratio between proliferating and hypertrophic chondrocytes remained constant [66,84]. These results were supported by the study of Martin *et al.* [85], wherein leptin stimulated the growth of the femur and midshaft cortical area, independently of peripheral IGF-1. A similar IGF-1-independent effect of leptin on bone growth and EGP length was observed in our food-restricted animals immediately after food replenishment [47], as reported also by others [86] and in IUGR rats [87]. The tibial CU growth was preceded by weight gain associated with an increase in serum leptin level [7]. Leptin administration restored the fasting-induced drop in GH secretion [75,88], but failed to increase serum IGF-1 levels [66,89]. Thus, leptin directly stimulates EGP cartilage proliferation and differentiation [66,79,80,81,82,83,84,85,86,87,88,89,90,91] through its active, long-form receptor [66,67,79]. This effect might be exerted via activation of the PTHrP/Ihh axis [84].

### 2.4. Glucocorticoids

Glucocorticoid level is increased under stressful conditions, such as acute or chronic diseases and prolonged food restriction. Glucocorticoids affect growth directly, by binding to their receptors on the EGP or indirectly via other endocrine signals. Numerous clinical studies have reported growth attenuation in children with chronic inflammatory diseases treated with prednisone and dexamethasone, synthetic glucocorticoids [92,93,94]. Although the primary disease and the associated inflammatory cytokines secreted may also have played a role in these cases by suppressing the activity of the GH/IGF-1 axis [95], studies in animal models and cell cultures reported a direct negative effect of dexamethasone on growth. In primary cell culture, a stimulatory effect on proteoglycan synthesis was noted at low concentrations of glucocorticoids with a suppressive effect at high concentrations [96]. Glucocorticoids above physiological levels inhibited EGP proliferation [97]. Furthermore, analyses of fetal rat metatarsal bone in organ culture [98] and the chondrocyte ATDC5 cells [99] showed a suppressive effect of dexamethasone on proliferation and hypertrophy, as well as a downregulation of GHR, IGF-1 and IGF-1R expression [100]. Thus, increased circulating levels of glucocorticoids may provide another explanation for the resistance of the EGP to GH and IGF-1 at the receptor level during food restriction [101]. In addition, hepatic IGFBP-1 mRNA and serum IGFBP-1 were significantly elevated by dexamethasone, suggesting an additional mechanism, whereby glucocorticoid excess leads to growth inhibition [102].

### 2.5. Thyroid Hormone

Hormones produced by the thyroid gland, 3,3′,5-triiodothyronine (T3) and thyroxine (T4), regulate metabolism. They also play an important role in skeletal maturation. The enzyme type II iodothyronine deiodinase (DIO2) activates thyroid hormone by converting the pro-hormone T4 to the bioactive T3. This conversion can take place also locally, in the EGP. By binding directly to the thyroid hormone receptors, TRa1, TRa2 and TRb1, in resting and proliferating chondrocytes, thyroid hormone inhibits chondrocyte proliferation and stimulates differentiation, mineralization and angiogenesis [103]. Deletion of TRa1, but not TRb, results in stunted growth, disorganized growth plate columns, delayed hypertrophy and delayed cartilage mineralization [104]. Children with hypothyroidism exhibit slow longitudinal bone growth, culminating in growth failure, whereas children with thyrotoxicosis have advanced skeletal maturation. Nevertheless, both conditions eventually lead to short stature. In several food restriction models, it was shown that the level of T3 was significantly reduced, while concentrations of T4 were generally left unaltered, suggesting that food restriction affects the conversion of T4 to T3 (mice fed 60% of their normal amount of chow for 12 weeks [105], rats fed 60% of their normal amount of chow for 4.5 months [106] or rhesus monkeys fed 70% of their normal amount of chow for more than 10 years [107,108]). As T3 seems to stimulate the recruitment of cells to the proliferating zone from the germinal zone and to facilitate the differentiation of growth plate chondrocytes, this reduction may contribute to growth attenuation.

In both animal and clinical studies, hypothyroidism was associated with a reduced height of all layers of the EGP and EGP disorganization. In a study performed on hypothyroid young rats, it was shown that the decline in the rate of longitudinal bone growth was delayed, and there was a reduction in EGP senescence, enabling CU growth [18]. Thyroid hormones are also essential for normal deposition of the ECM. They stimulate the production of type II and type X collagens and the synthesis of alkaline phosphatase, as well as IGF-1 and cytokines. The EGP of hypothyroid rats also has abnormal cartilage matrix deposition, with an abnormal increase in sulfation of heparan sulfate (HS) proteoglycans in proliferating chondrocytes. This abnormal matrix is deposited in a patchy irregular fashion, suggesting that thyroid hormones influence ECM biology, as well as the cellular activity of the EGP. As HS is required for binding of FGF to FGFR and for ligand-induced receptor activity, as well as for Ihh activity, T3-regulated production of HS, or the modification of its structure, might be one of the mechanisms by which T3 regulates EGP growth [109].

### 2.6. FGF-21

The fibroblast growth factor (FGF) family exerts broad mitogenic and cell-survival activities and is involved in a variety of biological processes, including embryonic development, cell growth, morphogenesis, tissue repair, tumor growth and tumor invasion. Most of the FGF ligands are restricted to close interactions due to their binding to heparin or HS in the ECM. However, a distinct group of FGFs (FGF-19, FGF-21 and FGF-23) lacks the conventional heparin-binding domains and binds heparin relatively poorly. They are readily present in the circulation and function in an endocrine-like manner [110], exerting pleiotropic effects on distant tissues and playing critical roles in the metabolic network. FGF-21, a 21-amino acid polypeptide, is abundantly expressed in the liver, pancreas, adipose tissue and muscle. It caught the attention of endocrinologists, when it was shown to improve glucose, insulin and triglyceride levels in diabetic mice, and it is now considered a metabolic hormone. Its overexpression in transgenic mice resulted in a lean, insulin-sensitive phenotype [111,112]. The levels of FGF-21 vary in response to the nutritional status [113,114]. On the one hand, high levels of FGF-21 have been reported in obese children [115] and youth [116], particularly those with a fatty liver. They correlated significantly with the adiposity index, the body mass index-standard deviation score and leptin level and increased with oral glucose load [117]. Similarly, in animal studies, plasma FGF-21 levels were significantly increased in mice and monkeys fed a high-fat diet compared to controls [118,119]. On the other hand, FGF-21 levels were increased by prolonged fasting in mice [120,121] and in extreme fasting conditions (for seven days) in humans [122], while moderate weight loss had no effect [123].

FGF-21 and two of its receptors, FGFR1 and FGFR3, as well as β-klotho, a co-receptor required for FGF-21-mediated receptor binding and activation, are all expressed by EGP chondrocytes [124]. Transgenic mice overexpressing FGF-21 exhibited reduced bone growth, high levels of GH and significantly low levels of IGF-1 compared with wild-type mice [125]. In a series of elegant experiments, Wu *et al.* [124] showed that high concentrations of FGF-21 directly suppressed EGP chondrocyte proliferation and type X collagen (Col10A1) gene expression. It also caused GH insensitivity through induction of the leptin receptor overlapping transcript (LEPROT) and transcript-like proteins [124], which could explain the EGP resistance to GH and IGF-1 at the receptor level after food restriction. The latter finding of Wu *et al.* [126] is supported by an earlier transcriptomic analysis suggesting that FGF-21 acts primarily by blunting GH/IGF-1 signaling in the liver [114].

Our experiments showed that FGF-21 inhibits the proliferation of the chondrocytes cell line, ATDC5 (unpublished observation), in accord with the results of Wu *et al.* [124]. However, the serum FGF-21 level significantly decreased in the food-restricted animals compared to animals in which the food restriction was lifted and those fed *ad libitum*. This finding was supported by western blot analysis showing a lesser production of FGF-21 in the liver and EGP [48]. These results are in line with the study of Hondares [127], wherein elevated FGF-21 mRNA levels were maintained in mice fed a high-fat diet and decreased in fasted neonates, but contrary to the study of Wu *et al.* [124]. The differences between the studies showing increased levels of FGF-21 following prolonged fasting in mice and the reduction in its level following ten days of food restriction in rats may be attributed to differences in the species used and in the duration and extent of the food-restriction protocol.

### 2.7. Vitamin D

Vitamin D can be obtained from dietary sources or synthesized in the skin by photo-conversion of 7-dehydrocholesterol in response to sun light and is stored in fat tissue. Its levels will be therefore reduced by prolonged food restriction.

To achieve biological activity, this compound must undergo two major consecutive modifications. First, it is metabolized in the liver to produce 25 hydroxyl-vitamin D2 (25OHD, calcidiol); next, it is converted in the kidney (or other tissues, including bone) by 1-α hydroxylase to generate the active form, 1,25 hydroxy-vitamin D3 (1,25(OH)2D3) (1,25OHD; calcitriol), which is the principal hormonal form of vitamin D. In addition, it can be converted to 24R,25 dihydroxy-vitamin D3 (24R,25(OH)2D3; 24R,25OHD), which was recently shown to have also specific biological effects, in both chondrocytes and osteoblasts [128,129]. Vitamin D is known to be involved in calcium homeostasis: it mediates the absorption or reabsorption of calcium in the intestine, bone and kidneys. Vitamin D regulates endochondral ossification in a cell-maturation-dependent manner via the nuclear vitamin D receptor (VDR), as well as a membrane-associated 1α,25 (OH)2D3-binding protein, ERp60 (protein disulfide isomerase A3) [130]. Both resting zone and growth-zone cells possess enzymes involved in the metabolism of 25OHD, and they produce and secrete the 24R,25OHD metabolite and the 1,25OHD metabolite into their extracellular environment [129]. Cells in the resting zone respond primarily to 24R,25OHD, and cells in the prehypertrophic and upper hypertrophic zones respond primarily to 1,25OHD [131]. The cell maturation-specific actions of the metabolites affects ECM synthesis and turnover, including matrix composition with the release and activation of latent factors, such as TGF-β, all of which fine-tune the rate and extent of chondrocyte proliferation and hypertrophy [4]. Recent studies have also shown that 24R,25OHD is essential for osteoblasts differentiation from mesenchymal stem cells and that it increased the expression levels of alkaline phosphatase, osteocalcin and osteopontin in the osteoblasts [128,132].

Interestingly, in the absence of vitamin D or in the presence of a malfunctioning receptor, rickets rather than short stature is described. However, several association studies that have dealt with single-nucleotide polymorphisms (SNPs) in the coding and intronic regions of the human VDR gene and numerous diseases showed a clinical association between SNPs located upstream of the transcriptional start site of the main human VDR gene promoter and height [133,134,135]. Loss of function mutations in the VDR do not interfere with embryogenesis and fetal development in humans or mice, and newborns appear normal at birth. However, after weaning, their rate of weight gain and linear growth, as well as serum calcium and phosphate levels decline. In VDR^−/−^ mice, bone mineralization is decreased and EGP cartilage is disorganized, with irregular columns of chondrocytes and increased matrix [136]. Analysis of VDR or 1-α null mice revealed phenotypic abnormalities, characteristics of vitamin D-dependent rickets, with decreased bone mineralization, growth retardation and aberrant EGP development after weaning. Impaired apoptosis of hypertrophic chondrocytes, with ordinary proliferation, was demonstrated to cause the significant widening and disorganization of the EGP [137]. These phenotypes could largely be rescued by dietary supplementation with calcium and phosphate, in agreement with the primary systemic role of calcitriol in intestinal calcium absorption.

In pediatric patients, diminished renal function is commonly accompanied by a disturbed bone metabolism and reduced linear growth. This was attributed in part to the reduced food consumption due to reduced appetite, increased level of glucocorticoids due to inflammation and also to impaired metabolism of vitamin D.

## 3. Local Molecular Mechanisms in Malnutrition and CU Growth

Several interrelated energy-regulatory mechanisms/factors in cells may be important for linear growth. These include, among others, microRNAs (miRNAs), transcription factors (e.g., hypoxia-inducible factor 1α, HIF1α), energy-sensing enzyme involved in translation (like mammalian target of rapamycin, mTOR), autophagy and epigenetic regulation (like histones modification). All have been shown to be significantly affected by food restriction and re-feeding.

### 3.1. microRNAs

On completion of the Genome Project, researchers recognized that of the approximately three billion nucleotides identified, only 2% are transcribed to protein-coding genes. The non-coding, previously considered “junk”, DNA has recently been found to be highly relevant to the regulation of gene expression and the maintenance of genomic stability. Some of it is transcribed into long non-protein-coding RNAs (lncRNA) or small non-protein-coding RNAs, or microRNAs (miRNAs). miRNAs are transcribed by RNA polymerase II-producing primary (pri)-miRNAs, which vary greatly in size, from a few hundred bases to tens of thousands [138]. Mature miRNAs, measuring approximately 19–23 nucleotides in length, negatively regulate the expression of a large portion of the protein-coding and non-protein-coding genes at the post-transcriptional level. Each miRNA can regulate one to several mRNA transcripts, and conversely, a single mRNA may be regulated by one to several miRNA sequences [139,140]. Computational analysis indicates that as many as 50,000 miRNAs may exist in the genome, and an estimated 33% of all mammalian mRNAs is regulated by miRNAs.

Mature miRNAs are derived from two major processing events driven by sequential cleavages by the RNase-III enzymes, Drosha, in the nucleus, and Dicer, in the cytosol. They are guided to their target following their incorporation into the RNA-induced silencing complex (RISC). This is accomplished by base-pairing of the mRNAs with their complementary miRNA binding sites, most of which are thought to lie in the 3' untranslated region (UTR) of the target. In mammals, miRNAs have been shown to regulate numerous systems, including adipocyte differentiation [141], insulin secretion, β-cell development [142], neural stem cell fate, immune function and cellular metabolism [143]. miRNA dysregulation is associated with numerous diseases, including diabetes [144,145], cancer and neurodegenerative diseases [146].

The central role of miRNAs in mammalian development was first reported by Bernstein *et al.* [147], who found that development was arrested at E7.5 in mice devoid of Dicer. In addition, mice lacking Dicer in their cartilage had many skeletal defects during development [148]. Dicer has been found to significantly affect limb size and morphogenesis, and its absence leads to a delay in the expression of limb-patterning genes [149]. When a high-throughput miRNA microarray technology was used to identify the battery of miRNAs that are expressed in the mature EGP, the most highly expressed was miR-140, the chondrocyte-specific miRNA [150,151]. Additional miRNAs shown to be important for chondrocyte regulation were as follows: miR-365, which mediates the effect of mechanical loading on chondrocyte proliferation and differentiation by targeting the histone deacetylase (HDAC) 4 [152]; miR-199a, a bone morphogenetic protein (BMP)-2 responsive miRNA that adversely regulates early chondrocyte differentiation by targeting the transcription factor, Smad1 [153]; and Let-7, the largest miRNA species in chondrocytes [148], which is important for chondrocyte proliferation. Let-7 and miR-140 coordinately regulate skeletal development [154].

We have found that some of the miRNAs expressed in the EGP of mature animals respond to nutritional cues. Furthermore, we have shown a direct link between miR-140, miR-22 and SIRT1 in the EGP [21]. SIRT1 is an HDAC of the sirtuin family (see the section on epigenetics). Binding sites for miRNA-140 were predicted in numerous genes known to play a role in chondrogenesis, such as vascular endothelial growth factor (VEGF)-A, matrix metalloproteinase 13 (MMP13), basic FGF2, platelet-derived growth factor (PDGF) receptor [143,155,156], HDAC4 [151] and a disintegrin and metalloproteinase with thrombospondin motifs (ADAMTS)-5 [157]. Conserved binding sites for miR-22 were described on numerous genes, including HDAC4 [158,159], SIRT1 [21,160], P21 [161] and others that are correlated with cell growth and transcriptional regulation. Relatively less conserved targets include genes implicated in developmental and metabolic processes, like HIF-1α and BMP7 [162]. Food restriction reduced the levels of miR-140 and miR-22, thus relieving the inhibition on the translation of SIRT1, leading to an increase in SIRT1 protein level [21]. Interestingly, although both SIRT1 and HDAC4 were reported to be targets of miR-22 [158,159], in our study, there was no change in HDAC4 level during food restriction, and the level of HIF-1α was reduced.

In our model, only a few miRNAs were reduced by food restriction, and none were significantly increased. With the exception of miR-140, none of the miRNAs identified were previously reported to be associated with chondrocyte function [150,151,154,155,163,164,165]. Although these miRNAs were found in both the proliferative and hypertrophic zones of the EGP, it was mainly the levels in the proliferative zone that were affected by nutritional manipulation. This finding is in line with earlier findings of a severe reduction in cell number, zone height and BrdU-positive cells in the proliferative zone during food restriction [47]. Together, they may suggest that miRNAs are associated with cell proliferation. Indeed, miR-21 and miR-126 were previously reported to be involved in growth regulation [166,167].

We have recently shown that miRNA expression is sensitive to serum derived from nutrition-manipulated animals. Specifically, all four miRNAs that were reduced by food restriction *in vivo* [21] were reduced by serum from food-restricted rats *in vitro*. Furthermore, when incubated in serum from rats with CU growth, they were not different from the control [48]. The addition of leptin and IGF-1 in physiological concentrations to the serum from the food-restricted rats partially reversed the effect of food restriction on miRNA levels, supporting the role of miRNAs in the nutrition growth control.

### 3.2. HIF1α

Studies have consistently found that food restriction affects energy metabolism and cell growth, regulation of transcription and stress and immune functions [168], but they failed to identify a common gene across the various species and tissues examined. In our rat model of food restriction, we observed dramatic changes in the expression of several genes, including some coding for transcription factors. One of them was hypoxia-inducible factor 1α (HIF-1α), a key subunit of the transcription factor, HIF. HIF-1α levels were reduced by food restriction and rapidly increased on re-feeding [7].

Under normoxic conditions, HIF-1α is rapidly degraded by the ubiquitin proteasome pathway. The process is mediated by the interaction of HIF-1α with the von Hippel–Lindau protein (pVHL). This interaction is triggered by the post-translational hydroxylation of proline residues via prolyl hydroxylase, located on the oxygen-dependent degradation domain (ODDD). Under hypoxic conditions, HIF-1α is stabilized and serves as a master transcription factor regulating the expression of several genes that code for proteins involved in angiogenesis, cell metabolism, proliferation, motility, adhesion and survival [7].

HIF-1α is responsible for the adaptation of chondrocytes to the low-oxygen pressure of the avascular and relatively hypoxic tissue in which they are located. Its significance to chondrocyte survival, especially in the hypoxic regions of the embryonic EGP, and its involvement in chondrocyte proliferation, differentiation and growth arrest are well recognized [169,170]. HIF-1α is expressed in the entire region of developing chondrocytes; its ablation results in embryonic lethality, with massive chondrocyte apoptosis. It was also found to be a major factor in anaerobic glycolysis, which supplies most of the energy requirements of the chondrocytes. In addition, it plays a regulatory role in ECM production by upregulating the expression of the cartilage transcription factor, Sox9, and by regulating the enzymes responsible for the hydroxylation of collagen prolines (P4HaI and P4HaII) and lysyl oxidase, an enzyme responsible for the formation of cross-links between collagen molecules [171]. Studies have shown that the stability and transcription activity of HIF-1α is enhanced by mammalian Runt-related transcription factor (Runx2), which promotes the expression of a number of chondrogenic and osteogenic markers, such as type I collagen, osteopontin and osteocalcin, via its interaction with ODDD and competition with pVHL to inhibit ubiquitination [172]. This, in turn, stimulates the expression of VEGF, the transcription of which is activated by HIF-1α, even under normoxic regions, and encourages the invasion of micro-vessels during endochondral bone formation.

In our rat model, HIF-1α was reduced by food restriction and increased during CU growth, probably to meet the increased needs for energy by the rapidly growing EGP [7]. This effect was demonstrated already after one day of re-feeding, concomitant with the increase in EGP height. Our findings indicate that nutrition has a profound effect on the level of gene expression within the EGP during longitudinal growth and that transcription factors, such as HIF-1α, play important roles in the growth of the mature EGP in response to nutritional manipulation [7].

### 3.3. Autophagy (Compound Recycling)

Autophagy is a catabolic process that results in autophagosome-dependent lysosomal degradation of bulk cytoplasmic content, abnormal protein aggregates and an excess of damaged organelles. Prenatally, it may be a part of the normal development of organs and plays a role in the control of several physiological processes [173]. It occurs continuously at basal levels during the homeostatic turn-over of cytoplasmic components required to meet metabolic demands. Postnatally, it is induced mostly by stress stimuli, especially under energy-restricted environmental conditions, and is inhibited by nutrient sufficiency. Cells degrade the cytosolic content by the formation of a double-walled vesicular structure that eventually fuses with lysosomes. In this manner, energy and building blocks can be generated from the cells’ own protein and lipid stores. Nutrient-stimulated activation of mTOR leads to the phosphorylation and inactivation of components of the autophagy pathway [174]. This process, recently shown in normal EGP [175,176], may be associated with nutrition-induced regulation of growth attenuation.

Several miRNAs target autophagy genes and autophagy, and autophagy, in turn, regulates the biogenesis of miRNAs (reviewed in [177]). One of the miRNAs involved is miR-21, found to be reduced in our system, supposedly leading to enhanced autophagy and reduced growth [178]. Thus, nutrient insufficiency may increase the autophagic response in the EGP chondrocytes, reducing the size of the cells and EGP and leading to growth attenuation. When the restriction is short, this process may be reversible, but when it is prolonged, cell number may be reduced and growth stunted. Autophagy also plays a role during cellular senescence via degradation of aggregate-prone proteins and damaged organelles. Senescence may be caused by inappropriate removal of damaged intracellular components through autophagic degradation, suggesting an association between autophagy and CU growth.

### 3.4. Mammalian Target of Rapamycin (mTOR)

**Figure 3:**
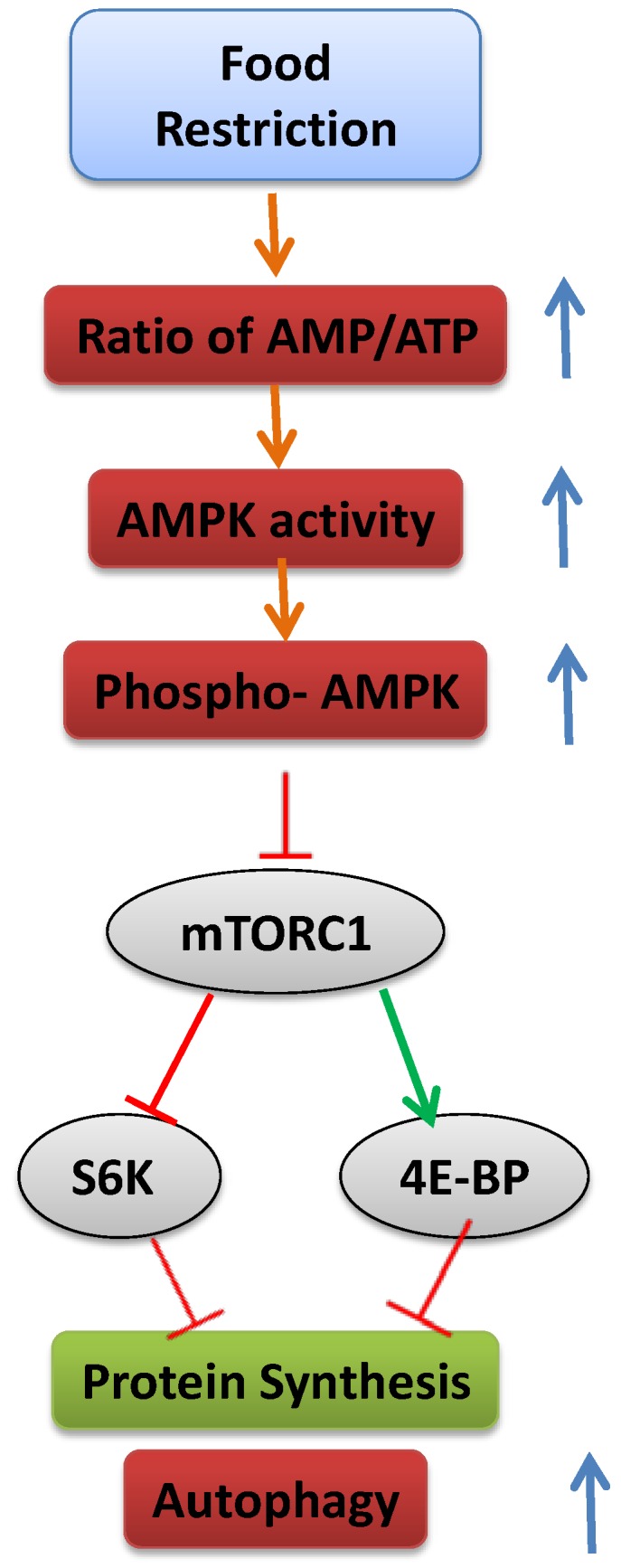
Schematic representation of the mechanism leading to inhibition of mTOR and activation of autophagy.

Cells have a complex sensing system to ensure that they do not grow in the absence of available nutrients that supply the energy to support that growth, including glucose, amino acids, lipoproteins and minerals. The cells’ translational machinery for the synthesis of proteins is activated by mTOR, an evolutionarily conserved serine/threonine protein kinase [179]. Activated mTOR stimulates angiogenesis, which increases the number of blood vessels through which nutrients can reach the cell. In addition, it increases the production of nutrient transporter proteins that enhance the cell’s ability to import essential nutrients and stimulates HIF-1α production and glycolysis. When nutrient levels are inadequate, mTOR is inactivated, protein synthesis is inhibited, cell growth is arrested and autophagic protein degradation takes place (see later) (Figure 3).

mTOR is found in the form of two multiprotein complexes, mTOR Complex 1 (mTORC1) [180,181] and mTOR Complex 2 (TORC2) [182,183]. mTORC1 is sensitive to the cellular nutritional state, and it targets the phosphorylation of proteins that regulate protein translation, gene expression and autophagy [184]. mTORC2 does not respond to changes in nutritional conditions, but is apparently involved in cytoskeleton regulation [182,183]. Two of the most-studied substrates of mTORC1 are eukaryotic initiation factor 4E binding protein (4E-BP) and ribosomal protein S6 kinase (S6K) [185]. 4E-BP is a translational inhibitor that is deactivated by mTORC1 phosphorylation; S6K is a positive translational effector activated upon phosphorylation. By inhibiting 4E-BP and activating ribosomal S6K, mTOR initiates translation [186,187].

mTORC1 is regulated by insulin and nutrients, including glucose and amino acids, particularly leucine, as well as a variety of cellular stresses. In some cell types, it is activated by amino acids alone; in others, amino acids collaborate with growth factors, such as insulin [188]. In the absence of amino acids, the growth factors are helpless. The specific amino acids necessary to activate mTORC1 are currently unknown. Moreover, the place at which they are first detected is still elusive, although the current model suggests that mTORC1 activation occurs at the lysosome and is mediated through an amino acid sensing cascade involving RAG, GTPase, Ragulator and vacuolar ATPase [189].

mTOR signaling has been shown to stimulate chondrocyte differentiation. Infusion of the mTOR inhibitor, rapamycin, into tibial bone inhibited proteoglycan accumulation and collagen X expression and decreased the level of Ihh [190]. The autophagic phase exhibited by hypertrophic chondrocytes [175,176] was shown to be dependent on the activities of mTOR together with HIF-1α and AMP kinase in response to the increased AMP/ATP ratio in the cells [191]. In an *in vitro* system, when AMP kinase or HIF-1α activity were blocked, autophagy could not be activated [192]. Autophagy was shown to be mediated by HIF-1α and its target gene, BNIP3, also in cancer cells [193]. However, in our system, food restriction led to reduced expression of both HIF-1α and several of its target genes, including BNIP3 [7], suggesting that the interactions between mTOR and HIF1α are dependent on the specific system.

### 3.5. Epigenetics

Epigenetics is defined as changes in gene function caused by mechanisms other than changes in the genomic DNA sequence, for example chromatin structure remodeling together with chemical modifications of DNA and associated proteins, such as histones. An easy way to imagine the role of epigenetics on gene expression is by making an analogy to music notes (DNA) and music instructions on a note sheet that describes the pitch, rhythm and tempo of a melody (epigenetics).

The term “histone code” is now widely used to describe the complex pattern of phosphorylation, acetylation, methylation, SUMOylation and possibly ubiquitination of histones and their impact on the expression of individual genes. Histone acetylation by histone acetyltransferases (HATs) occurs at the ε-amino group of the lysine residue within the peptide chain. The already bulky lysine side chain becomes even bulkier; the positive charge is neutralized, and the histone-DNA interaction is weakened. Acetylation is cleared by HDACs. As a general rule, histone acetylation by HATs is usually associated with increased transcription activity, because of the “loose” chromatin structure. Deacetylation by HDACs leads to condensation and suppression of transcription. The acetylation-deacetylation process may be the basis for the cyclic transcriptional re-initiation that occurs when a swift response to the oscillation of environmental stimuli is necessary. HATs and HDACs also use non-histone protein substrates, including transcriptional regulators, chromatin components and signaling factors, adding another level of regulation [145,194,195]. There are 18 HDACs in the mammalian genome, divided into four groups according to their homology to yeast HDACs, their size, cellular localization, catalytic domain and mechanism of action. There are 11 zinc-dependent HDACs—class I (HDAC1, -2, -3 and -8); class II, subdivided into class IIa (HDAC4, -5, -7 and 9) and class IIb (HDAC6 and -10); and class IV (HDAC11) [196,197,198]—and seven nicotinamide adenine dinucleotide (NAD+)-dependent HDACs, which comprise class III (sirtuin 1 to -7). Class I HDACs are generally located in the nucleus and are relatively small in size; class II HDACs are present both in the nucleus and cytoplasm and are generally larger. Several HDACs were shown to be stimulated by food restriction; others were shown to be affected by a high-fat diet [199], indicating their sensitivity to the metabolic status and suggesting a possible role in the nutrition-growth connection.

Several HDACs have been found to contribute to growth regulation. In one study, the addition of a pan-HDAC inhibitor to primary chondrocyte culture stimulated the expression of SOX9, an ECM cartilage regulatory gene, and induced histone acetylation at the collagen type 2a1 (Col2a1) gene enhancer [200]. Further evidence of epigenetic control of chondrocyte function was provided by a study showing that overexpression of HDAC1 or HDAC2 in chondrocytes resulted in downregulation of aggrecan and Col2a1 gene expression [201]. Knockout mice for HDAC3 were smaller than normal, and HDAC3-deficient chondrocytes were smaller than normal chondrocytes, entered hypertrophy sooner and produced lower levels of ECM proteins [202]; mTOR was suggested to be repressed in chondrocytes lacking HDAC3. HDAC4 was shown to be a potent inhibitor of the expression and activity of transcription factor RUNX2, which is essential for both chondrocyte and osteoblast differentiation. HDAC4 knockout mice developed ectopic chondrocyte hypertrophy, and overexpression of HDAC4 in proliferating chondrocytes inhibited chondrocyte hypertrophy and differentiation [151].

Several HDACs are stimulated by food restriction, pointing to their possible role in the nutrition-growth connection. The most-studied HDACs in this context are the sirtuins, which are highly-conserved enzymes that utilize NAD+ to deacetylate a number of histone and non-histone substrates. The founding member of this family, silent information regulator 2 (Sir2), promotes longevity in yeast by repressing gene expression and stabilizing chromatin. Mammals have seven Sir2 homologues (SIRT1–SIRT7) that are involved in regulating cell survival and stress response. SIRT1 and SIRT6 are involved in the response to food restriction [203,204]. Interestingly, they share functional similarity in terms of anti-senescence. Studies found that SIRT1 was induced by nutrient deprivation *in vitro* and after long-term food restriction *in vivo*. Cells cultured in the presence of serum from food-restricted rats showed an attenuation of stress-induced apoptosis and an increase in SIRT1 expression. The enzymatic activity of SIRT1 is positively regulated by NAD+, which increases during food restriction and fasting. Mice overexpressing SIRT1 exhibited similar physiological properties to mice on a food-restricted regimen, and SIRT1 knockout rodents were small [205] and had a deranged EGP (our unpublished observations). SIRT1 may regulate cell proliferation, senescence and apoptosis via several transcription factors that govern metabolism and endocrine signaling, including PPAR-γ [206], PGC-1α [207], FOXOs [208,209] and p53 [210]. It has also been shown to stimulate calorie restriction-induced autophagy [211] in the nucleus, as indicated by the ability of SIRT1 to interact with several autophagy genes (ATG5, ATG7, LC3 and FOXO). SIRT1 has the same effect in the cytosol, as indicated by its ability to regulate autophagy upstream of mTOR by binding to the mTOR inhibitor, TSC2 [212]. SIRT1 has been reported to activate autophagy by deacetylating several essential components of the autophagy machinery [213]. In addition, SIRT1 deacetylates HIF1α and reduces its activity [214]. It may also have a systemic effect on growth through its effect on GH secretion. This assumption is based on findings that resveratrol, a SIRT1 activator, significantly reduced GHRH-induced GH secretion from rat anterior pituitary cells *in vivo* and *in vitro* [215].

We have recently found that SIRT1 levels were increased in the EGP in food-restricted rats, followed by a rapid reduction on re-feeding, concomitant with the transcriptional activation [21]. Yamamoto [216] reported that after a 48-h fast, SIRT1 knockout mice had a higher level of IGF-1 in serum and Igf1 mRNA in the liver compared to fasting wild-type mice. The author suggested that SIRT1 mediates GH resistance in states of under-nutrition, probably by de-acetylating a lysine residue in signal transducer and activator of transcription 5 (STAT5) that is important for the GH signal transduction mechanism [216]. Interestingly, SIRT1, was reported to stimulate IGFBP-1 promoter activity through a FoxO-dependent and independent mechanism [217], and SIRT1 overexpression was show to reduce the basal and IGF-1-induced collagen I gene expression [218], thus providing another way to inhibit skeletal growth.

SIRT6 is the only known sirtuin whose absence in mice causes genome instability and the premature appearance of aging-related pathologies [219]. Levels of SIRT6 were also found to increase in rats subjected to prolonged food restriction, in mice after 24 h of fasting and in cell culture after nutrient depletion [204]. SIRT6 knockout mice showed reduced body size, and neural-specific SIRT6 knockout mice showed postnatal growth restriction due to reduced GH and IGF-1 levels [220]. The SIRT6 protein is highly and preferentially expressed by the EGP in proliferating and prehypertrophic chondrocytes. Accordingly, in SIRT6 knockout mice, the height of each of the different zones of the EGP and the primary spongiosa are reduced. Studies by Piao *et al.* [221] pointed to the role of Ihh activation in SIRT6 action on cell proliferation. SIRT6 binds to the Ihh promoter in primary chondrocytes; thus, Ihh is a downstream target of SIRT6 in chondrogenesis. SIRT6 knockout mice have an increased expression of intracellular adhesion molecule 1 (ICAM-1) and plasminogen activator inhibitor 1 (PAI-1), leading to increased senescence. SIRT6 inhibits IGF-Akt-mTOR signaling through the suppression of IGF signaling-related genes [222], leading to autophagy [223], and it functions as an HIF-1α co-repressor at promoters of HIFα glycolytic target genes via HDAC-activated deacetylation of H3K9 [224], important in both autophagy and senescence. We found that the expression (mRNA) and level (protein) of SIRT6 were increased in the EGP of food-restricted animals and rapidly returned to baseline on food replenishment (unpublished observations). These results are in line with reduced HIF1α transcription activity [7].

## 4. Conclusions

**Figure 4:**
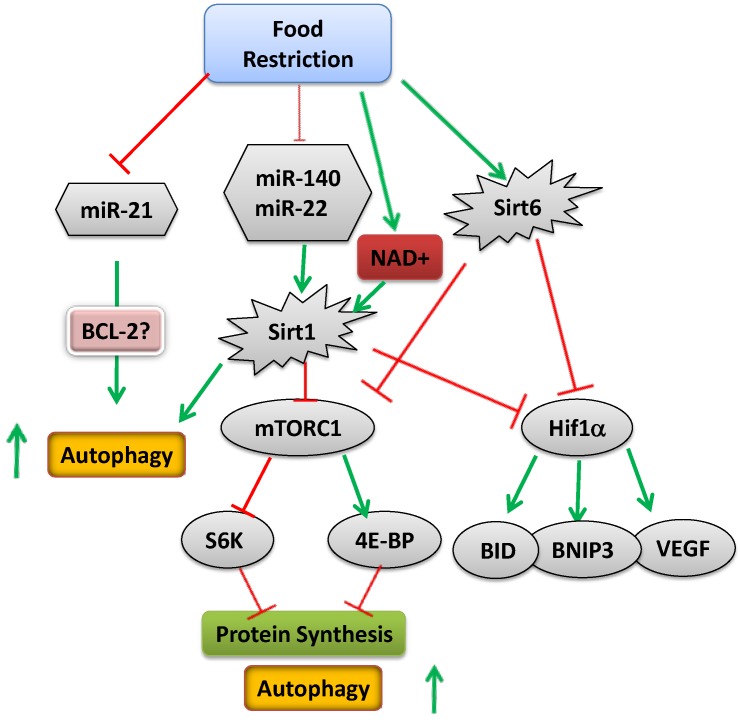
Schematic representation of the mechanism activated upon food restriction (green arrows indicate increased activity or level; red arrows indicate reduced activity or level).

Common knowledge is based on the wisdom of hundreds of generations. It is clear that malnutrition impairs linear growth; the exact mechanism by which the body signals the EGP of the long bones to grow or attenuate growth is still unclear, although several possible mediators are beginning to emerge. Regulation takes place at multiple levels, including systemic factors, like hormones, and local factors, including miRNAs, transcription factors, enzymes (mTOR) and epigenetic mechanisms, all affected in response to nutritional cues (Figure 4). Deciphering the role of epigenetic regulation in growth may open a new era of research and pave the way for the development of new treatments for children with growth disorders.

Metabolic control over chondrocyte growth is apparently mediated, at least in part, by serum components, including leptin and IGF-1. Other factors affected by nutritional status, such as thyroid hormone, GH, cortisol, insulin, IGFBP-1 and 7 and FGF-21, may also be involved. Our findings also show a role for local (intrinsic) factors to the growth attenuation, as well. The change in specific miRNAs and HDACs in response to the metabolic state, both *in vivo* and *in vitro*, suggests that they play an important role in the nutrition-growth link.

As CU growth returns individuals to their genetic growth trajectory, it is usually considered a success of medical treatment. However, over the past two decades, it has been increasingly recognized that rapid infant CU growth markedly increases the risk of adult health disorders, particularly metabolic syndrome, obesity, cardiovascular disease, as well as adverse pulmonary, renal and cerebral function [225]. Recently, we have shown that CU growth may also have deleterious effect on bone quality, at least in the short term [226]. Studying the molecular mechanisms regulating CU growth may lead to the establishment of better nutritional and therapeutic regimens for more effective and safer CU growth in children with malnutrition and growth abnormalities. It will be fascinating to follow this research in the coming years and to translate the knowledge gained to clinical benefit. When mothers tell their children to eat in order to grow properly, they are right, but we still do not know exactly why.

## Abbreviations Used in this Review (in Alphabetical Order)

ATG: autophagy-related genes
BMP: bone morphogenetic protein
CU: catch-up
ECM: extracellular matrix
EGP: epiphyseal growth plate
FGF: fibroblast growth factor
GH: growth hormone
GHR: growth hormone receptor
HAT: histone acetyl transferase
HDAC: histone deacetylase
HIF: hypoxia inducible factor
IGF-1: insulin-like growth factor 1
IGF-1R: insulin like growth factor 1 receptor
IGFBP: insulin-like growth factor binding protein
Ihh: Indian hedgehog
IUGR: intrauterine growth retardation
LC3: microtubule-associated protein light chain 3
lncRNA: long non-coding RNA
miRNA: microRNA
mTOR: mammalian target of rapamycin
ODDD: oxygen-dependent degradation domain
PThrP: parathyroid hormone-related peptide
SGA: small for gestational age
UTR: untranslated region
